# Long-term survival in permanent middle cerebral artery occlusion: a model of malignant stroke in rats

**DOI:** 10.1038/srep28401

**Published:** 2016-06-22

**Authors:** Nagesh C. Shanbhag, Robert H. Henning, Lothar Schilling

**Affiliations:** 1 Division of Neurosurgical Research, Medical Faculty Mannheim, Heidelberg University, Mannheim, D-68167, Germany; 2 Department of Clinical Pharmacy & Pharmacology, University Medical Center Groningen, University of Groningen, 9700 RB, The Netherlands

## Abstract

Occlusion of the middle cerebral artery (MCA) by an intraluminal filament is widely used to study focal brain ischemia in male Sprague-Dawley rats. However, permanent occlusion goes along with a high fatality. To overcome this drawback we designed a new filament carrying a bowling pin-shaped tip (BP-tip) and compared this with three conventionally tipped filaments. Follow-up periods were 24 h (all groups) and 72 and 120 h in BP-tip group. Ischemic damage and swelling were quantified using silver nitrate staining. Collateral flow via the posterior cerebral artery (PCA) was assessed using selective dye perfusion of the internal carotid artery. Despite a comparable decrease of brain perfusion in all groups, ischemic damage was significantly smaller in BP-tips (p < 0.05). Moreover, BP-tip significantly reduced mortality from 60% to 12.5% and widely spared the occipital region and hypothalamus from ischemic damage. Conventional but not BP-tip filaments induced vascular distortion, measured as gross displacement of the MCA origin, which correlated with occipital infarction size. Accordingly, BP-tip occluded rats showed a significantly better collateral filling of the PCA territory. Ischemic volume significantly increased in BP-tip occlusion at 72 h follow-up. BP-tip filaments offer superior survival in permanent MCA occlusion, while mimicking the course of a malignant stroke in patients.

Stroke is the most common cerebrovascular disease and a major cause of death and disability worldwide. In 2005 the estimated global incidence was approximately 70 per 100,000^1^, which will increase steadily with expected 7.8 million cases by 2030^2^.

Approximately 85% of all stroke cases are ischemic and present as neuronal dysfunction due to a critical reduction of cerebral blood flow in a circumscribed part of the brain^3^. The pathophysiology of tissue damage in brain ischemia is highly complex and despite extensive basic and clinical research not yet fully understood. Moreover, there is a long-standing translational roadblock, i.e. a clinical failure of successful experimental therapeutic advances^4^. The reasons for this block are manyfold and may include drawbacks of the models used.

Currently the most widely used focal brain ischemia model is the intravascular filament occlusion of the middle cerebral artery (MCA) in rodents originally developed in rat^5^. This method has repeatedly been modified, particularly by using a silicone cover of the filament, and we successfully used it to study the role of early hypoxia in ischemic damage^6^,^7^. The intravascular filament occlusion method is preferred as its removal at any desired time allows superior control of the size of ischemic damage. However, such model may not reflect permanent occlusion, as the pathophysiological course is likely affected by the reperfusion injury. Indeed, a 6 h MCA filament occlusion (MCAO) results in less damage than a 3 h occlusion period followed by 3 h of reperfusion^8^. However, the experimental benefit of re-establishing blood flow, notably the improved survival of animals, usually exceeds the drawbacks of reperfusion injury. Unfortunately, therapeutic thrombolysis to enable reperfusion is possible only in the minority of patients, usually <10% due to the restricted time window of up to 4.5 h after onset of symptoms. Thus, a large number of stroke patients may be considered to present a kind of permanent occlusion condition during the acute phase. In fact, a review of cerebral angiography studies has yielded evidence that approximately 50% of stroke patients display vessel occlusion 3–4 days after onset of symptoms^9^. Previously, models of thrombus-induced focal ischemia have been advocated to reproduce ischemic stroke in patients best. However, this experimental approach has severe disadvantages including partial or complete resorption of the thrombus^10^. Other permanent ischemia models include craniectomy often followed by surgical or photothrombotic occlusion distally to the MCA origin generally resulting in less severe ischemic damage. Moreover, they result in large variation of the infarct size due to regional differences between occlusion sites and reduced edema compared to filament occlusion.

Unfortunately, permanent filament occlusion produces a high mortality as described upon its introduction^11^. The huge territorial infarction and the considerable mortality rate in filament-based permanent occlusion are important features of the clinical picture termed malignant stroke which is a devastating disease pattern encountered in up to 10% of all stroke patients^12^,^13^. While neuroprotective therapies have frequently produced a significant reduction in filament-induced brain infarction following transient MCAO, such protective effects were consistently absent in permanent focal ischemia^14^,^15^,^16^. Thus, an experimental model to mimic malignant stroke without the ethically unacceptable significant mortality rate is highly warranted. We, therefore, set out to improve permanent MCAO (pMCAO) in rat by designing a totally new shape of occluding tip. The results indicate that our new method strongly reduces mortality, thus reliably extending the studies over days or even weeks, enabling the study of mechanisms of tissue damage, edema resolution and tissue remodeling. Its major features include a huge territorial infarction, marked brain edema and a delayed growth of ischemic damage over several days, suggesting that the method can well be considered a reliable and clinically relevant model of malignant stroke.

## Results

### Survival after 24 h of pMCAO

Two animals (one each in L-TB and S-TB groups) were excluded from all analysis because of occurrence of a subarachnoid haemorrhage upon filament insertion identified in the LDF traces as described previously^17^. These animals were not allowed to recover from anesthesia, and post-mortem examination confirmed the bleed in both instances. Mortality rates in the different experimental groups during the 24 h after successful pMCAO were 0% (DOC group), 12.5% (BP-group), 54% (L-TB group), and 65% (S-TB group). An overall significant difference in mortality was observed [χ^2^(3) = 11.44, n = 44, p = 0.01]. Due to the absence of mortality in DOC group, we could only compare significance level of differences between the other 3 groups (p < 0.05, BP vs S-TB) (Table 1). Further, volumetric analysis of the ischemic damage was performed in animals which survived the full 24 h observation period. To balance the number of surviving animals, the overall numbers of animals included in each experimental group differ markedly (Table 1). Moreover, L-TB and S-TB animals surviving the 24 h presented in a very bad clinical condition which would not appear compatible with an extended survival period. Similarly, 4 out of the 6 animals in the DOC group showed severe worsening of their clinical state when approaching the end of the 24 h observation period. In contrast, rats which underwent MCAO with a BP-type filament were in markedly better clinical condition, which was considered compatible with an extended survival period. Failure of occlusion based on LDF reduction was not encountered in any of the groups.

### Ischemic damage 24 h post pMCAO

**Figure 1:**
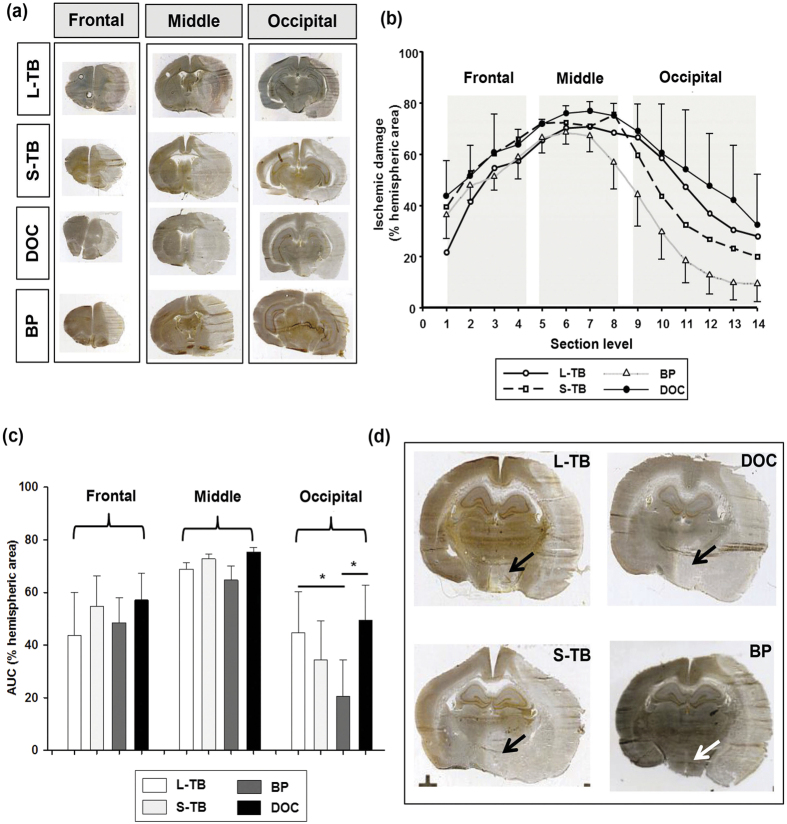
Ischemic damage after 24 h of permanent middle cerebral artery occlusion. (**a**) Representative brain sections from frontal, middle, and occipital regions, (**b**) hemispheric infarction and (**c**) the area under curve (AUC) analysis in the different experimental groups. The sections were grouped into a frontal, middle, and occipital region along the fronto-occipital axis (with section 7 assigned to the bregma for reference). (**d**) Representative slices depicting hypothalamic involvement (arrows) of infarction in different groups (bregma, −3.14 mm). L-TB tip, long tubing-based silicone coated filament tip; S-TB tip, short tubing-based silicone coated filament tip; DOC, commercially available filaments (Doccol); BP, bowling pin-shaped filament tip. ^*^p < 0.05, BP 24 h occipital vs L-TB, DOC occipital levels. Data are expressed as mean ± SD. Statistical significance was determined by a one-way ANOVA with Tukey’s test.

The LDF signal measured over the MCA territory decreased by about 65% during introduction of the filaments without differences between groups (Table 1). Nevertheless, BP-tip filaments induced significantly less ischemic damage than the other 3 types of filaments while brain swelling was similar in all 4 experimental groups (Table 1). Next, we examined the regional distribution of ischemic damage in the occluded hemispheres (Fig. 1a,b). The AUC of ischemic damage was similar for all 4 experimental groups in the frontal and middle regions, but BP-tip occluded animals had a significantly smaller volume in the occipital region (Fig. 1c; p < 0.05 vs. L-TB and DOC tip occluded rats). Also, BP-tip occluded animals showed a remarkably smaller infarct size in the region of the hypothalamus (mostly located in the section levels 9 to 11) compared to other groups (Fig. 1d).

### Mortality and ischemic damage in long-term BP-tip occluded rats

In view of the superior survival, good clinical condition and relatively restricted infarct area in BP-tip occluded animals the observation period was extended to analyse long-term effects. Animals were sacrificed after 72 h (n = 7) or 120 h (n = 6). In these groups no cases of premature death were encountered. Furthermore, there was no failure of occlusion. Ischemic volume significantly increased by approximately 40% from 24 h to 72 h (p < 0.05), followed by a decline at 120 h, when infarcted area amounted to 116% of the value measured after 24 h of ischemia (Table 2). Since all volumes were corrected for brain swelling, these data imply a distinct delayed growth of the infarction beyond 24 h of pMCAO, most prominent in the occipital region and less so in the frontal region. No difference was observed in the middle region, which is largely supplied by the MCA. In contrast, the decline of ischemic damage from 72 h to 120 h was comparable in all brain regions (frontal, middle, and occipital regions) without reaching statistical significance. The accompanying brain swelling, however, was comparable between 24 and 72 h post occlusion, but declined significantly at 120 h (Table 2).

### Morphology of brain after 24 h of pMCAO

**Figure 2:**
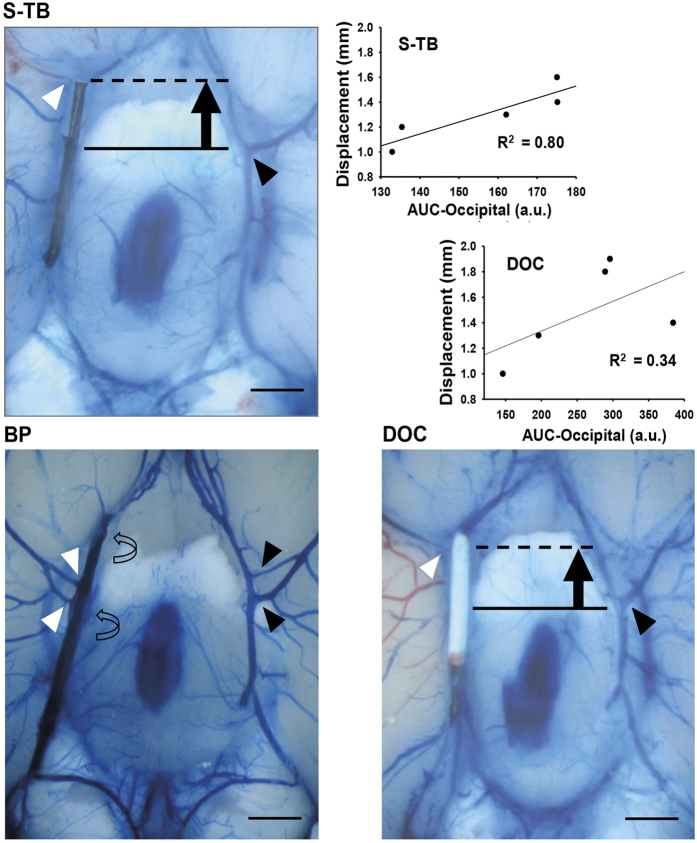
Intravascular filament occlusion of the middle cerebral artery (MCA) may lead to displacement of its origin. Depicted are examples in which a short tubing-based silicone coated (S-TB) filament tip, a commercially available filament (DOC) and a bowling pin-shaped (BP) filament tip were used. The origin of the right-sided MCA is indicated by a white arrow head (right side) and a black arrow head (left side). The proximal and distal ball-shaped thickenings of the BP filament are indicated by curved arrows. Note the split origin of the MCA in the case shown on the right. Such a deviation may occur in up to 10% of a given batch of rats. The overlay indicates how the degree of displacement (indicated by the length of the black arrow) was measured. This displacement shows a good correlation (top right panel) with ischemic damage (as calculated from AUC in this case) produced in the occipital part of the brain in S-TB and DOC-tip filament groups (n = 5 in each group). Scale bar = 1 mm. AUC, area under curve. Linear regression was conducted by least squares method.

The observation that, despite a comparable reduction of the LDF signal, the BP-tip occluded animals had a significantly smaller volume of ischemic damage at 24 h of pMCAO suggests factors other than the mere occlusion of the MCA to contribute. Therefore, we carefully inspected the brains after removal. The main striking finding was a gross vascular distortion displacing the origin of the MCA in L-TB, S-TB, and DOC tip filaments (Fig. 2). However, such a displacement was never observed in BP-tip occluded animals. Displacement quantified by calculating the rostral shift of the origin of the right versus left MCA (Fig. 2) amounted to 1.4 ± 0.4 and 1.3 ± 0.2 mm in S-TB and DOC tip occluded animals, respectively. Additionally, ischemic damage of the occipital part strongly correlated with the displacement in S-TB (r^2^ = 0.80) and DOC-tip (r^2^ = 0.34) (Fig. 2).

### Filament-induced alterations of the perfusion pattern

**Figure 3:**
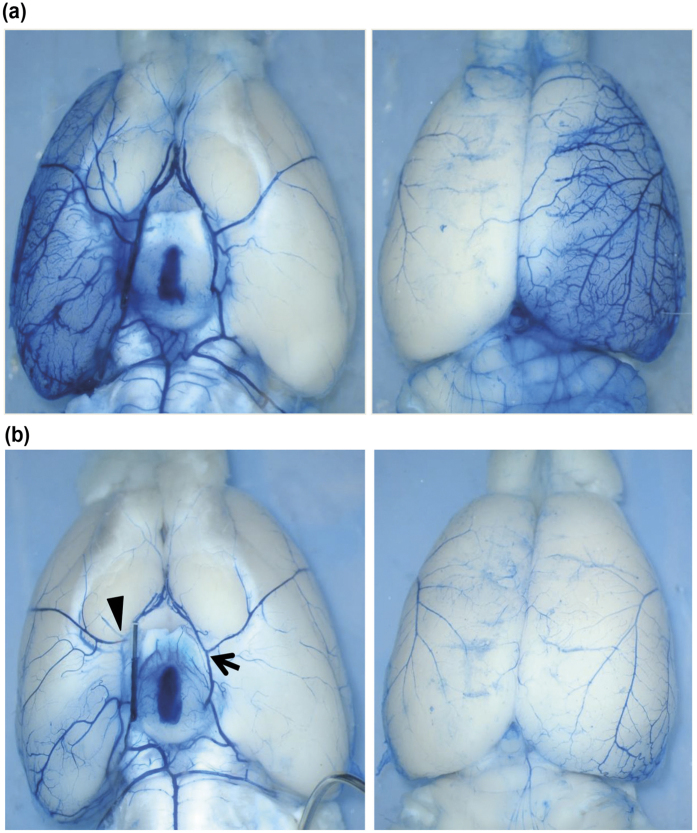
Vascular filling pattern after selective infusion of a small volume of physiological saline solution (PSS) containing 2% Evans blue (EB) into the internal carotid artery (ICA). The occluding filament was left *in situ* during the infusion. Depicted are examples in which a BP-tip filament (upper panel-a) or a S-TB tip filament (lower panel-b) were used for occlusion (the images exemplify the conditions found in 3 animals occluded with a BP-tip and 3 animals occluded with a S-TB tip). The pictures indicate the retrograde filling of the main trunk and daughter vessels of the middle cerebral artery (MCA) via collateral arteries fed from the posterior cerebral artery. The filling of the collateral system was considerably better in the BP-tip occluded animal. Some dye has also entered the main trunks of the cerebral arteries in the contralateral hemisphere via the posterior communicating arteries. Note the displacement of the MCA origin on the occluded side (arrow head) in the lower panel, in comparison to the contralateral side (arrow) at the base of brain. A detailed outline of the collateral pathways and EB filling pattern between MCA and posterior cerebral artery (PCA) is shown in Supplementary Figure S1.

In view of the vascular distortion at the base of the brain and the regional differences in the development of ischemic damage in the conventional groups, we wondered about a possible impairment of collateral blood flow through the posterior cerebral artery (PCA). To examine this, additional experiments used selective dye perfusion of the ICA with BP and S-TB occluding filaments *in situ* (n = 3 in each of the two study groups). We infused a small volume of 2% EB solution and employed a very low infusion speed to delineate the low resistance pathways fed by the internal carotid artery (ICA) and documented the distribution of the dye by microphotographs. Hemispheres from BP showed a markedly better filling of the superficial microvasculature than S-TB (Fig. 3a,b). This improved EB filling across the PCA territory in BP-tip filaments also revealed the pre-existing collateral vessels between the PCA and the MCA, easily identified by a cork-screw like appearance (a feature akin to arteriogenesis), likely providing a path for retrograde filling of the MCA daughter vessels and even its main trunk. Particularly, Supplementary Figure S1a shows PCA branches coursing over the occipital pole to anastomose with MCA branches. In contrast, the parenchymal filling was markedly less in S-TB-tip (Supplementary Figure S1) although the collateral anastomoses between the PCA and MCA branches were also evident. In agreement, significantly more dye accumulated in the occipital pole of BP-tip occluded animals (Fig. 3a) than in S-TB occluded animals [EB content (μg/g tissue), 58.7 ± 8.2 with BP-tip occlusion vs. 9.0 ± 2.1 in S-TB-tip occlusion; p < 0.001)]. Similarly, the residual hemisphere (excluding occipital pole) displayed a higher EB content in BP-tip occluded animals (62.3 ± 14.5 μg/g tissue) than in S-TB-tip occluded rats (6.1 ± 3.3 μg/g tissue, p < 0.05). Moreover, no differences in dye accumulation per hemispheric region between BP- and S-TB-tip occluded animals were observed in the contralateral occipital poles and residual hemispheres. Together, these data imply that the markedly impaired filling of occipital regions in S-TB-tip group most likely results from the observed vascular distortion (Fig. 3b and see Supplementary Figure S1).

## Discussion

The current work describes a major improvement of the intravascular filament based MCA occlusion technique, which allows long-term survival in rats without reperfusion despite a large territorial infarction and marked edema. This important improvement is brought about by designing a completely new tip of the occluding filament. Selective perfusion studies demonstrate that this type of filament preserves collateral blood supply, mainly to the occipital part of the brain via the PCA. The apparent improvement of collateral blood supply delays the expansion of ischemic cell death so its maximum is reached only around 72 h after establishing MCA occlusion. Therefore, our new model nicely reflects the main features of malignant stroke in patients but significantly reduces its high mortality rate. The model may well provide a powerful tool to study the mechanisms of infarct progression and tissue remodelling and eventually test novel therapeutic strategies.

The intravascular filament occlusion of the MCA in rats is the most widely used stroke model, compromising the brain region most often affected in stroke patients. The advantages of intravascular filament occlusion have been discussed repeatedly^10^,^18^, and the most notable points include (i) maintenance of the intact skull bone, (ii) ease of establishing reperfusion, (iii) control of the infarct size by modifying the duration of occlusion, and (iv) high reproducibility of the tissue injury generated. In previous studies employing 2 h of MCAO followed by 6 or 22 h of reperfusion, we found substantial ischemic damage with <20% coefficient of variation with an overall mortality rate of <10%. However, changing to pMCAO will result in a high mortality rate as described since the earliest reports^11^, which has not improved dramatically since. Therefore, we designed a completely new shape of the silicone cover at the end of the filament resembling a bowling pin. With this type of filament, long-term survival was achieved with a low mortality rate even without reperfusion. We have examined the characteristics of this new method and found the degree of LDF decline upon pMCAO similar to 3 conventionally prepared albeit different types of filaments. Nevertheless, BP-tip filaments resulted in a significantly smaller ischemic lesion after 24 h of pMCAO, predominantly due to decreased occipital infarction. The latter indicates a major role of blood supply through the PCA, which in rodent brain originates from the intracranial part of the ICA^19^. While one may expect blockade of the PCA origin by long-tipped filaments, this is not the case for short-tipped filaments. However, scrutinizing the base of the brain consistently revealed distortion of the ICA and displacement of the MCA origin in all of our experiments with conventional tips, independent of the length of their silicone cover. With the special design of the new BP-type filament such distortion and displacement were not observed, most probably because the smaller diameter at the tip (distal bulge) allowed the filament to smoothly enter the anterior cerebral artery trunk. The functional importance of arterial displacement is reflected by the significant correlation between the degree of MCA origin displacement and the volume of ischemic damage in the posterior part of the brain, indicating the distortion to impair PCA blood flow. To address this hypothesis, measurement of blood flow in the occipital cortex may be considered, e.g. by laser speckle methodology. Unfortunately, this method is restricted to a more or less plane surface such as somatosensory cortex in the temporo-parietal region of the rat brain^20^,^21^,^22^. Thus we decided to study vascular filling over PCA and MCA supplied territories by selective perfusion of the ICA with a tracer dye with the occluding filaments in place and quantify the intraparenchymal dye content. We intentionally infused a small volume of EB-containing solution at a low speed in order to delineate low resistance pathways. This approach markedly differs from previously used protocols in rats in which a tracer was systemically injected with high pressure to identify even the smallest arteries on the base of the brain using μCT methodology^23^. Employing this technique, animals occluded with the newly designed BP-tip filament displayed a strikingly better filling with the dye of the superficial pial arteries in the occipital and the adjacent temporo-parietal region compared to short-tipped filaments. Moreover, improved filling of the pial vasculature also resulted in a significantly higher accumulation of the dye in the brain tissue. In contrast, the parenchymal filling was markedly less intense in S-TB-tip group, despite evident collateral anastomoses between the PCA and MCA branches (Supplementary Figure S1). Thus, the impaired filling of the posterior vascular bed in groups with conventional filaments is explained well by an obstruction of PCA inflow by the observed vascular distortion. Furthermore, PCA inflow obstruction appears also to reduce collateral blood supply to the posterior boundary zone of the ischemic core. The functional importance of collateral pathways has received increasing attention in the assessment of stroke patients recently^24^, and it is also evident in the present experimental study.

The difference in maintenance of collateral circulation was apparently of functional importance since the new BP-type filament not only resulted in a significantly smaller volume of ischemic damage 24 h after pMCAO, but also induced markedly lesser damage in the hypothalamic region. In fact, the hypothalamus in the rat receives blood supply from several sources including the PCA. Largely sparing the hypothalamus from ischemic damage with the new BP-tip filaments likely contributed substantially to the improved survival since the hypothalamus is of key importance in the regulation of vital physiological functions such as body temperature. In fact, hypothalamic damage has widely been related to early postischemic hyperthermia and occasionally considered an even inevitable consequence of filament-induced brain ischemia^25^. However, studies with telemetric or repeated measurement of body temperature after filament-induced MCA occlusion have challenged the validity of this dogma^26^,^27^,^28^. On the other hand it is well established that hyperthermia aggravates ischemic brain damage in models of global and focal ischemia of transient and permanent nature^25^,^26^,^29^. Similarly, in patients with ischemic stroke the occurrence of hyperthermia in the early ischemic phase correlates with a poor outcome^30^. Although we did not measure body temperature over an extended period of time, our data suggest that the extensive protection of the hypothalamic region might well have contributed to the lower mortality rate in BP filament group.

Rats undergoing MCA occlusion with our new BP-type filament presented in a markedly better clinical condition, allowing for extension of survival to 3 and even 5 days without premature deaths. In these experiments, the total volume of ischemic damage increased significantly from 24 to 72 h survival, particularly due to expansion in the occipital region supplied by the PCA. Apparently, the cells in the frontal and occipital brain areas were viable for an extended period after MCA occlusion before undergoing irreversible damage. Further studies to address the mechanisms behind this spread of cell death from the core region are highly desired because its mechanisms may well differ from those operating in the acute phase. Such studies may also help to overcome the long-standing translational road-block in stroke research. In addition, our new method will also allow studies on the mechanisms of trauma reorganisation and tissue remodelling in permanent ischemia, both anatomically and functionally.

In conclusion, the model of pMCAO using our new BP-tip filament results in a condition which closely resembles malignant stroke in patients. The main features include the large ischemic damage to the MCA territory, pronounced brain edema, and the significant lesion expansion due to delayed ischemic cell death. The BP-tipped filament does not compromise PCA blood flow, thus maintaining collateral blood supply of the occipital brain region including the hypothalamus and limiting mortality rate. For the first time we can now mimic malignant stroke in a well-established experimental setting, which allows for long-term follow-up studies to better characterize the pathophysiology of and explore interventional strategies in this clinically devastating disease.

## Material and Methods

### Animals

Adult male Sprague-Dawley rats (Janvier; Isle St. Genest, France) were kept under standardized housing conditions with free access to standard chow and water for at least one week before start of the experiment. All experimental methods were carried out in accordance with relevant guidelines and regulations and approved by appropriate licensing committee (Regional Council, Karlsruhe, Germany).

### Study Groups

**Figure 4:**
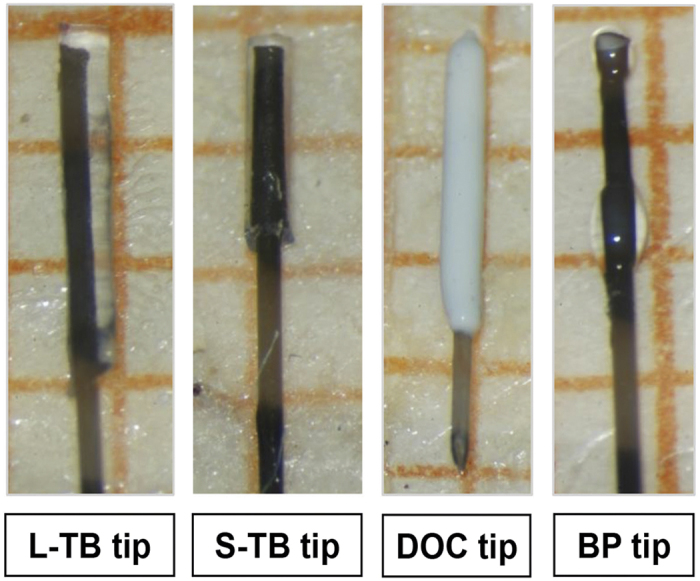
Filament types used in the present study. All filaments are shown on graph paper (edge length, 1 mm) for illustration of the tip size. The shape and the length of the tip coverage can clearly be distinguished. L-TB, long silicon length tubing-based; tip S-TB, short silicon length tubing-based tip; DOC, commercially available filaments (Doccol); BP, bowling pin-shaped silicon tip.

A total of 65 animals were used and randomised in various study groups. The animals (body weight 300–350 g) aged between 10–12 weeks underwent intraluminal filament occlusion of the MCA (MCAO) using different types of filaments. Custom-made filaments consisted of a nylon suture (Monosof, 4-0, Covidien, Neustad/Donau, Germany) with silicone tips as described below and compared to commercially available filaments (MCAO sutures, Doccol Corp., Sharon, MA, USA). Four types of filaments with effective occluding diameters of 390–425 μm were used (Fig. 4). To obtain similar numbers of animals surviving the procedure, rats were randomized to groups based on the prior amount of surviving animals to match experiments in time. All the filaments under different conditions were assayed in parallel.

#### L-TB type

Long tubing-based silicone coated (L-TB) filament. Coating of the tip was performed according to Spratt and co-workers^31^. Briefly, a polyethylene (PE) tubing was filled with a silicone rubber (Elastomer 43, Wacker Chemie Burghausen, Germany) and the suture introduced into the tubing. After polymerization the tubing was removed and the silicone coating cut to a length of approximately 3 mm.

#### S-TB type

Short tubing-based silicone coated (S-TB) filament. These filaments were prepared similarly to L-TB with a silicone tip length <2 mm.

#### DOC type

Filaments with the shortest tip available (2–3 mm in length) were obtained from Doccol.

#### BP type

Bowling-pin shaped tip. These filaments carried two ball-shaped thickenings separated by approximately 1 mm with the smaller diameter at its tip. It thus resembles a bowling-pin with the smaller diameter ranging between 325–350 μm and the larger diameter between 390–425 μm. This filament design with a tip resembling a bowling pin offers several major advantages over conventionally prepared filaments: (i) the smaller diameter at the tip (distal bulge) allowed the filament to smoothly enter the anterior cerebral artery (ACA) trunk without any major distortion of the internal carotid artery and block this pathway and thus retrograde flow via anterior communicating artery while the proximal bulge with a larger diameter blocked the anterograde flow via the ipsilateral ICA. (ii) the occluding part made up by the proximal and the tip bulge is much shorter which selectively blocks MCA origin. With its shorter layout the occluding tip completely passes the origin of the PCA from the internal carotid artery thus leaving this artery open. A schematic representation of creating BP type filament is described in Supplementary Figure S2.

In initial experiments, animals were sacrificed 24 h after pMCAO. In a further series of experiments, BP tip occluded animals were sacrificed 72 h and 120 h after pMCAO. In a third series, animals underwent selective perfusion with Evans blue (EB) dye with the filament left in place as described below.

### Surgical procedure

Right-sided pMCAO was produced as previously described^11^ with some modifications. The completeness of occlusion was checked by laser Doppler flowmetry (LDF; Perimed Instruments, Jarfalla, Sweden) with the probe positioned in the MCA supply territory (coordinates: 4 mm lateral and 2 mm posterior to the bregma). The pMCAO was induced by inserting the filament into the proximal external carotid artery and advancing it via the ICA to the origin of the MCA. In our group proper positioning of a filament is required to result in a drop of the LDF by >50% of the starting level, and animals which did not meet this value are excluded and were not allowed to recover from anesthesia. Filaments were left *in situ* for relevant time points (see Supplementary Information).

### Selective perfusion of Evans Blue

A total of 6 animals (n = 3 in each of the two study groups, BP and S-TB types) were used in this perfusion study. The experiments in this group were terminated 15 min after MCA occlusion. The animals underwent *in situ* perfusion with physiological saline solution (PSS) only. Then, the right-sided common carotid artery (CCA) was cannulated with a PE tubing (inner/outer diameter, 0.4/0.8 mm; RCT; Heidelberg, Germany) filled with 2% EB in PSS. Prior to cannulation, the pterygopalatine artery was completely ligated to allow maximum flow across the ICA. The catheter was inserted carefully in order to not affect the position of the filament fixed in the ECA stump. The dye was then infused at a very slow rate, 70 μl/min for 2 minutes using a LSP1 infusion pump (Hugo Sachs Electronic, March-Hugstetten, Germany). After perfusion the catheter was removed, the brain isolated and photographed for documenting the distribution of the dye in the pial vasculature. The occipital poles of the right and left hemisphere were dissected and the remaining brain cut in the midline. The tissue samples were snap frozen in n-pentane and stored at −80 °C until spectrophotometric analysis of the EB content.

### Spectrophotometric assay of EB content

Brain samples were homogenised in 50% trichloroacetic (1:4 weight/weight) using an Ultraturrax homogenizer (Ika-Werk, Staufen, Germany) for 30 s and centrifuged at 10,000 rpm for 20 min at 4 °C (Heraeus Biofuge Stratos, Thermo Fisher Scientific, Dreieich, Germany). Supernatant was collected and absolute ethanol added at a volume ratio of 1:2 for occipital regions and 1:1 for the remaining samples. Absorbance of EB was measured at 615 nm (Infinite M200, Tecan, Mannedorf, Switzerland). Tissue content of EB was quantified with using a linear standard curve and expressed as micrograms per gram fresh tissue.

### Ischemic damage assessment

Brain damage was determined using silver nitrate staining of serial coronal slices and corrected for swelling accordingly^6^,^7^,^32^. Hemispheric infarction along the fronto-occipital axis was calculated for 3 regions (frontal, middle and occipital) as area under the curve (AUC; see Supplementary Information).

### Statistical analysis

Data are expressed as mean ± standard deviation. Statistical analysis was performed by one-way analysis of variance (ANOVA) and subsequent pairwise comparison using the Tukey-test. Mortality and MCA displacement were compared by Chi-square test and linear regression by least squares method. With either test p < 0.05 was considered statistically significant.

## Additional Information

**How to cite this article**: Shanbhag, N. C. *et al*. Long-term survival in permanent middle cerebral artery occlusion: a model of malignant stroke in rats. *Sci. Rep.* **6**, 28401; doi: 10.1038/srep28401 (2016).

## Supplementary Material

Supplementary Information

## Figures and Tables

**Table 1:** Laser Doppler flow reduction (upon occlusion of the middle cerebral artery origin), ischemic volume, hemispheric swelling and mortality in different filament groups at 24 h of permanent middle cerebral artery occlusion.

	Type of occluding filament			
	L-TB (n = 6)	S-TB (n = 6)	DOC (n = 6)	BP (n = 7)
LDF reduction (%)#	58.7 ± 8.9	60.9 ± 11.3	71.0 ± 5.7	67.7 ± 9.8
Ischemic volume (mm^3^)#	448.3 ± 78.3*	423.2 ± 50.6*	551.8 ± 113.8*	306.4 ± 30.0
Hemispheric swelling (%)#	35.9 ± 5.7	36.5 ± 7.9	37.1 ± 9.9	28.9 ± 7.4
Mortality (died/total no.)$	7/13	11/17	0/6^@^	1/8^**^
Total number of animals	• underwent permanent MCAO: 46			
	• not allowed to recover from anesthesia due to LDF traces indicative of subarachnoid hemorrhage during filament insertion: 2, excluded from all analyses			
	• allowed to recover from anesthesia but died before 24 h: 19, excluded from infarct analyses			
	• survived the 24 h period: 25, included in infarct analyses			

LDF, laser Doppler flow; L-TB, long tubing-based silicone coated filament tip; S-TB, short tubing-based silicone coated filament tip; DOC, commercially purchased filament (Doccol Corp., Sharon, MA, USA); BP, bowling pin-shaped filament tip. ^#^Data represent animals which survived the 24 h observation period. ^*^p < 0.05 vs. BP tip. The values of ischemic brain damage are corrected for swelling to improve comparison between the groups. Values are represented as mean ± SD. Statistical significance was determined by a one-way ANOVA with Tukey’s test. ^$^A significant association was observed between the groups and mortality [χ^2^(3) = 11.44, n = 44, p = 0.01] using Chi-Square test with Bonferroni correction. ^@^Between group comparison in DOC group is precluded by absence of deaths. ^**^p < 0.05 vs S-TB using between group comparison with Bonferroni correction. MCAO, middle cerebral artery occlusion.

**Table 2:** Time-related changes of ischemic damage in bowling pin-shaped tip occluded rats undergoing permanent middle cerebral artery occlusion.

BP group	24 h (n = 7)	72 h (n = 7)	120 h (n = 6)
Ischemic volume (mm^3^)	306.4 ± 30.0	434.1 ± 63.1*	355.9 ± 103.1
Hemispheric swelling (%)	28.9 ± 7.4	29.1 ± 4.8	13.2 ± 1.9**
AUC-Frontal region (%)	48.5 ± 9.4	55.5 ± 8.7	41.9 ± 9.5
AUC-Middle region (%)	64.7 ± 5.3	68.9 ± 3.6	62.7 ± 3.5
AUC-Occipital region (%)	20.1 ± 14.3	32.3 ± 15.5	22.4 ± 17.2

BP, bowling pin-shaped filament tip; AUC, area under the curve.

^*^p < 0.05 vs. 24 h, ^**^p < 0.001 vs 24 and 72 h. Values are represented as mean ± SD. Statistical significance was determined by a one-way ANOVA with Tukey’s test.
