# Supplementary material for: Long-term survival in permanent middle cerebral artery occlusion: a model of malignant stroke in rats
Source: Sci Rep. 2016 Jun 22;6:28401. doi: 10.1038/srep28401 (PMC4916503; doi:10.1038/srep28401)
Supplement: Supplementary Information [file srep28401-s1.pdf]

Long-term survival in permanent middle cerebral artery occlusion: a model of malignant stroke in rats

Nagesh C. Shanbhag, MBBS, MSc [1,2,*], Robert H. Henning, MD, PhD [2], Lothar Schilling, MD, PhD [1,*]

[1]Division of Neurosurgical Research, Medical Faculty Mannheim, Heidelberg University, Mannheim, D-68167, Germany

[2]Department of Clinical Pharmacy & Pharmacology, University Medical Center Groningen, University of Groningen, 9700 RB, The Netherlands

[*]Correspondence to:

Lothar Schilling (lothar.schilling@medma.uni-heidelberg.de)

Nagesh C. Shanbhag (n.c.shanbhag@umcg.nl)

SUPPLEMENTARY FIGURE 1

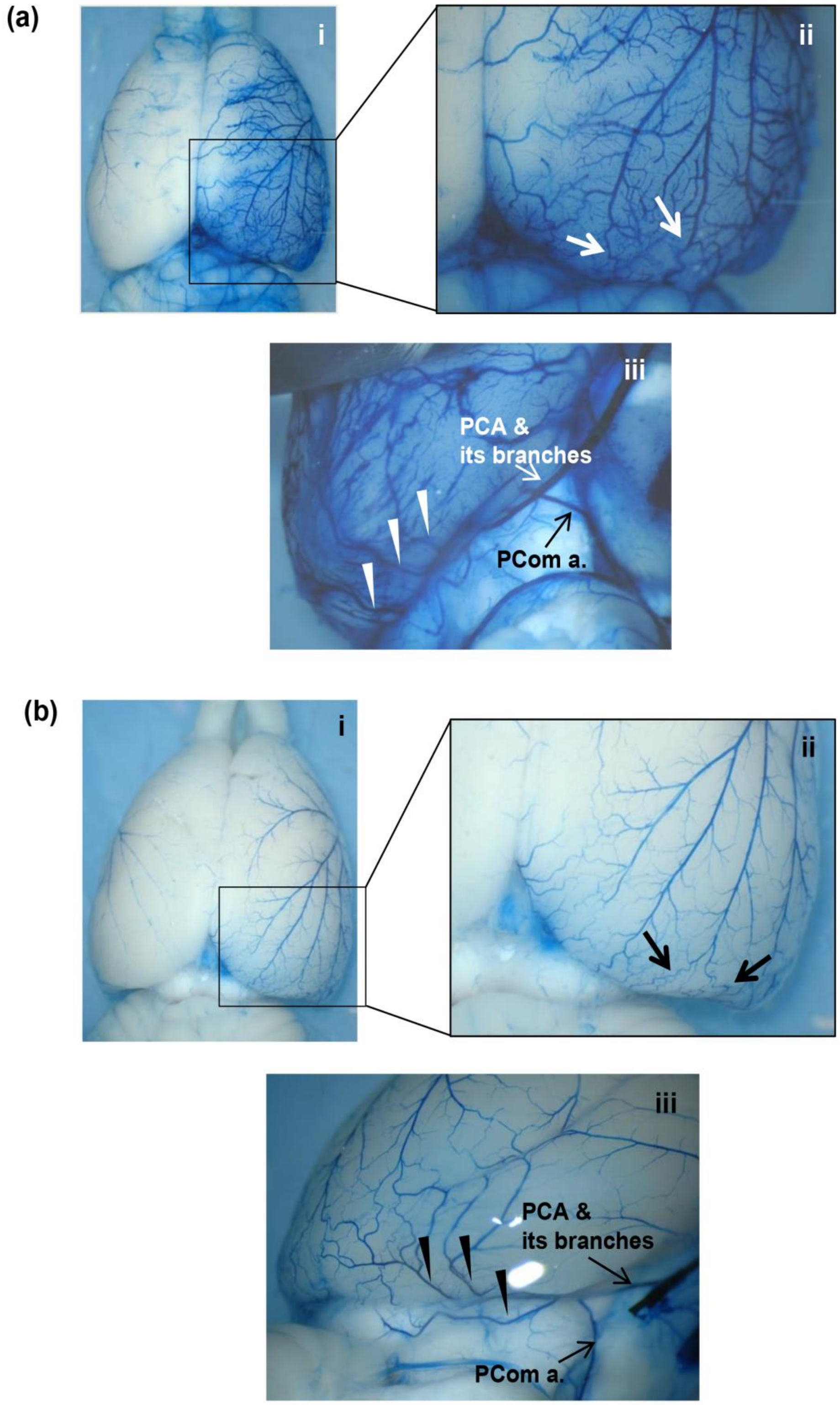

Figure S1. **Collateral pathways and dye filling pattern upon selective EB perfusion via CCA**. (a) With a BP-tip occluding filament *in situ*, MCA filling is observed to originate via the PCA and its branches forming collateral anastomoses with those of the MCA. The PCA

branches (iii, white arrow heads) course over the occipital pole towards the dorsal aspect of brain anastomosing with the MCA branches (ii, arrows). Intraparenchymal filling displayed high intensity in the occipital pole, and a similar degree of filling was also seen in the MCA supplied territory (i, ii). (b) In contrast, the vascular filling was markedly less intense in S-TB-tip filament occluded animals although the collateral anastomoses between the PCA and MCA branches were evident (ii, black arrows and iii, arrow heads). The less intense filling (i & ii) is most probably due to the vascular displacement occurring in S-TB-tip occlusion leading to an inflow obstruction in the PCA which was absent with BP-tip filaments. EB, Evans blue; CCA, common carotid artery; BP, bowling pin-shaped tip; MCA, middle cerebral artery; PCA, posterior cerebral artery; S-TB, short tubing-based tip.

SUPPLEMENTARY FIGURE 2

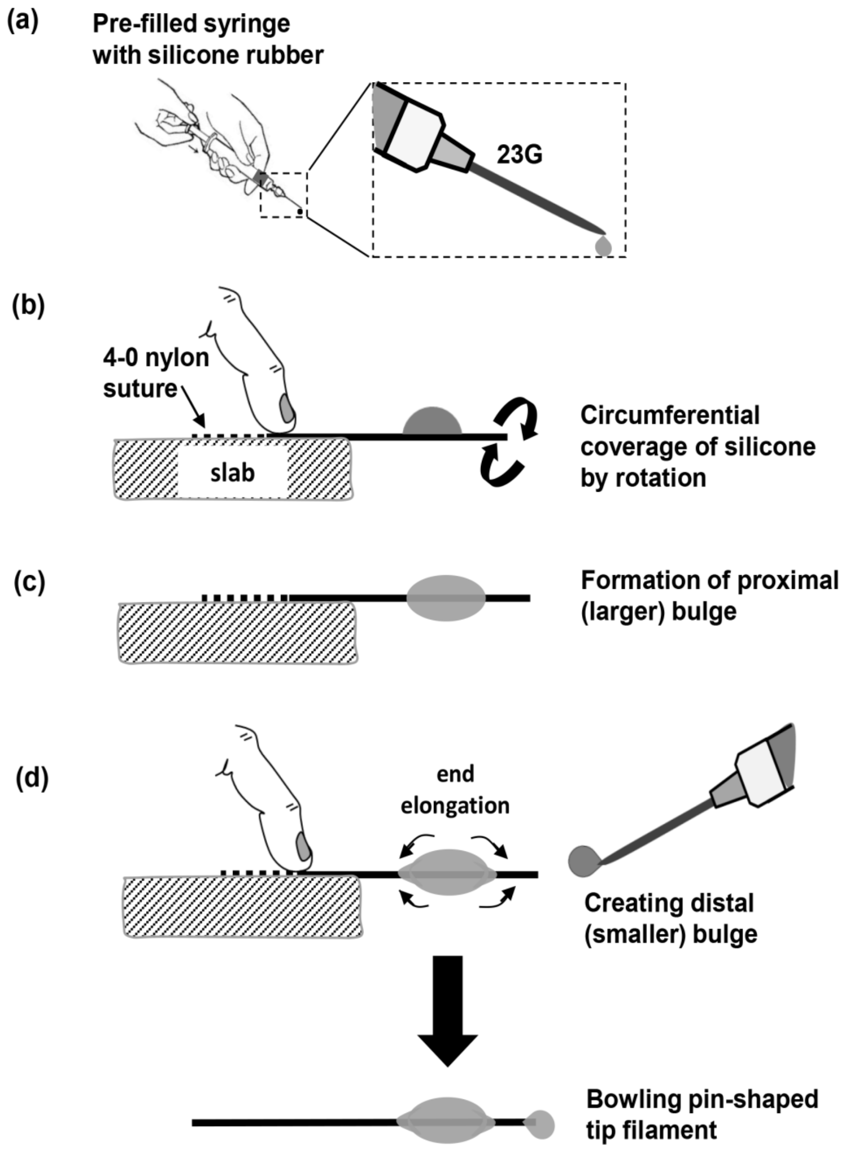

Figure S2. Schematic representation of creating a bowling pin-shaped silicone tip filament. The whole procedure is carried under the microscopic guidance with an ocular eyepiece coupled with a micrometer scale. (a) A 5 ml syringe equipped with a 23G syringe is filled with silicone rubber (Elastomer 43; Wacker Chemie, Burghausen, Germany). The plunger is gently pressed until a drop of silicone is ejected from the tip of needle (avoid air bubbles). (b) Meanwhile, a 4-0 nylon suture segment (~3 cm in length) is placed on a slab covered with a scale paper. The drop of silicone is transferred onto the nylon suture at approximately 1 mm from the tip of filament. Rotate the filament with the finger by gently

sliding on to the slab to enable the silicone drop to cover the filament circumferentially. The diameter is repeatedly controlled with the micrometer scale. This results in the formation of the proximal (larger) bulge (c) with the desired occluding diameter. (d) With a firm proximal grip over the filament, gently extend the ends of the proximal bulge for a firm fixation. This can be done using an additional syringe fitted with a 30 G needle. Thereafter, a relatively smaller drop of silicone is placed at the tip of the filament in order to create the distal (smaller) bulge. This is again done under repeated microscopic control. The resulting silicon coating resembles a bowling pin-shaped tip filament. The distance between the two bulges is approximately 1 mm. The filament is then air dried overnight to firmly fixate the silicon on to the suture surface. Each of the filament tips was measured to confirm the appropriate diameter and length before employing the filament for arterial occlusion. This schematic figure is created by NCS.

SUPPLEMENTARY METHODS

Induction of pMCAO

For induction of focal cerebral ischemia, we used the intraluminal middle cerebral artery (MCA) occlusion approach introduced by Longa and co-workers (1) with some modifications. Briefly, after a midline incision in the neck, the right common carotid artery (CCA) was dissected between the sternocleidomastoid and the sternohyoid muscles and the vagal nerve carefully disassociated from the adventitial sheath. The carotid bifurcation was exposed and the internal carotid artery (ICA) dissected. After transient occlusion of the CCA and the ICA by ligatures the external carotid artery (ECA) was permanently ligated distally to the origin of the superior thyroid artery. The ECA was cut next to the site of occlusion, a filament introduced and advanced into the ICA until a sharp drop of the LDF signal indicated occlusion of the MCA. After control of completeness of the occlusion and exclusion of intracranial bleeding (2), the filament was tightly fixed to the ECA. We deliberately chose LDF over laser speckle imaging for the following reasons: (i) laser speckle imaging requires extensive skull exploration meaning high surgical trauma which might further increase the ischemic trauma, and (ii) the brain surface over the occipital pole is not appropriately accessible and feasible for laser speckle imaging. Following MCA occlusion the animals received a subcutaneous injection of buprenorphine (3 µg/100 g body weight) for post-surgical pain control. Finally, the incisions in the neck and over the skull were closed after removal of the LDF fibre. Isoflurane was withdrawn and the animals were transferred to their home cage with free access to drinking water and moistened food pellets.

Termination of experiments

At the end of the observation period, the animals were deeply anesthetized. A large bore PE tubing was introduced into the abdominal aorta for *in situ* perfusion with physiological saline solution (PSS) upon severing of the inferior caval vein After 4 min perfusion was switched to PSS containing 1% Evans Blue (Sigma-Aldrich GmbH, Steinheim, Germany). For brain isolation, both the parieto-temporal skull bones were removed and the underlying dura dissected out, the animal turned supine and its head positioned with the dorsal surface facing downwards. Thereupon, we carefully cut the spinal cord near to the transition from the hindbrain using a scalpel. The cranial nerves and carotid arteries were cut as proximally as possible by means of a microscissor without exerting any force upon the vascular structures. This procedure resulted in the occluding portion of the filament resting within the ICA at the MCA origin without any artefactual displacement. Next, the brain was removed and photos were taken of the basal and dorsal aspects. The brain was then snap-frozen in n-pentane solution (Merck Schuchardt OHG, Hohenbrunn, Germany) and stored at -80°C.

Ischemic damage assessment

For volumetric analysis of brain damage serial coronal cryosections (thickness, 20µm) were taken at a distance of 1000µm (cryotome HM500, Microm GmbH, Wiesloch, Germany). The sections were air-dried and fixed in 4% paraformaldehyde for subsequent high contrast silver nitrate staining as developed by Vogel and coworkers (3) and used by our group previously (4,5). After staining, the slides were scanned using a flat-bed scanner (1640SU; Epson, Cologne, Germany). The areas of both hemispheres and of ischemic damage were outlined on the monitor and measured using ImageJ software (free-ware available under http://rsbweb.nih.gov/ij/docs/guide/index.html). The degree of hemispheric swelling was determined on each section level using the equation:

$$\textbf{Swelling}\ (\%) = 100\left(\frac{\text{volume}_{\text{ischemic hemisphere}} - \text{volume}_{\text{contralateral hemisphere}}}{\text{volume}_{\text{contralateral hemisphere}}}\right) \quad \text{eq. 1}$$

In order to improve comparison between the experimental groups ischemic damage was corrected for the actual degree of swelling using the equation (6),

$$\mathbf{Ischemic\ area_{corrected}} = \left(\frac{\mathrm{area_{ischemic\ region}}}{\mathrm{area_{ischemic\ hemisphere}}}\right)\left(\mathrm{area_{contralateral\ hemisphere}}\right) \quad \mathrm{eq.\,2}$$

In each animal, the volume of ischemic damage (in mm^3) was calculated by adding the individual ischemic areas for each section based on the results of the planimetric analyses and multiplying by the distance between the sections (1000µm). In addition, brain infarction size was also calculated for each section level in % of the respective hemispheric area. The sections were ordered from frontal to occipital and numbered consecutively. Using the level next to the bregma as reference (assignment based on the rat brain atlas by Paxinos and Watson (7) the sections were aligned to 3 regions along the fronto-occipital axis (i) frontal region, sections 1 – 4, (ii) mid region comprising sections 5 – 8, and (iii) occipital region comprising sections 9 – 14. An area under the curve (AUC) analysis was performed to determine the extent of ischemic damage in each of the 3 regions.

Bibliography of studies included in Supplemental methods

(1) Longa, E. Z., Weinstein, P. R., Carlson, S., Cummins, R. Reversible middle cerebral artery occlusion without craniectomy in rats. *Stroke*. **20,** 84-91 (1989).

(2) Woitzik, J., Schilling, L. Control of completeness and immediate detection of bleeding by a single laser-Doppler flow probe during intravascular middle cerebral artery occlusion in rats. *J. Neurosci. Meth.* **122,** 75-78 (2002).

(3) Vogel,. J., Möbius, C., Kuschinsky, W. Early Delineation of Ischemic Tissue in Rat Brain Cryosections by High-Contrast Staining. *Stroke,* **30,** 1134-1141 (1999).

(4) Woitzik, J., Weinzierl, N., Schilling, L. Early administration of a second generation perfluorochemical decreases ischemic brain damage in a model of permanent middle cerebral artery occlusion in the rat. *Neurol. Res*. **27,** 509-515 (2005).

(5) Seiffge, D. J. et al. Improvement of oxygen supply by an artificial carrier in combination with normobaric oxygenation decreases the volume of tissue hypoxia and tissue damage from transient focal cerebral ischemia. *Exp. Neurol*. **237,** 18-25 (2012).

(6) Liu, S., Zhen, G., Meloni, B. P., Campbell, K., Winn, H. R. Rodent Stroke Model Guidelines for Preclinical Stroke Trials (1st Edition). *J. Exp. Stroke Transl. Med*. **2,** 2-27 (2009).

(7) Paxinos, G., Watson, C. *The Rat Brain in stereotaxic coordinates 3rd edn,* (Academic Press, 1997).
